# Completed absorption of coronavirus disease 2019 (COVID-19) pneumonia lesions: a preliminary study

**DOI:** 10.7150/ijms.54675

**Published:** 2021-04-03

**Authors:** Xue-yan Liu, Fa-jin Lv, Sheng-xiu Lv, Bin-jie Fu, Wang-jia Li MD, Yu Ouyang, Zhi-gang Chu

**Affiliations:** 1 Department of Radiology, The First Affiliated Hospital of Chongqing Medical University, Chongqing, 400016, China.; 2 Department of Radiology, Chongqing Public Health Medical Center, Chongqing, 400036, China.

**Keywords:** Coronavirus Disease 2019, chest CT imaging, acute respiratory disease

## Abstract

**Background:** Complete absorption of coronavirus disease 2019 (COVID-19) pneumonia in a short term was not detailedly reported. We aimed to investigate the clinical and imaging characteristics of COVID-19 patients with complete absorption of pulmonary lesions.

**Methods:** Retrospectively collected the clinical and chest CT data of 224 patients with COVID-19 in one regional medical center. Currently, pulmonary lesions in 37 patients were completely absorbed. The clinical manifestations, laboratory examinations, and CT findings of lesions for these patients were summarized.

**Results:** Among the 37 patients (age, 39.0 ± 12.4 [14-63] years, 20 males), disease in 36 (97.3%) was mild and in 1 (2.7%) was from severe to mild. The most common symptoms were cough (24/37, 64.9%) and fever (23/37, 62.2%). Their laboratory indicators at admission were usually normal, while the white blood cell and neutrophil count significantly increased at discharge (p = 0.004, p = 0.006). On initial CT images, all patients had various pulmonary lesions (mean involved lobes: 2.8 ± 1.5, range: 1-5; mean involved segments: 6.6 ± 4.3, range: 1-16), which mainly manifested as multiple patchy and or spherical ground glass opacities (GGOs) (30/37, 81.1%) with fibrous strips (19/30, 63.3%) or consolidation (11/30, 36.7%). After treatment, lesions in most (33/37, 89.2%) patients were continuously absorbed. At discharge, previous lesions were mostly absorbed in 11 patients (11/37, 29.7%), the main residues were GGOs (24/37, 64.9%), followed by fibrous strips (13/37, 35.1%). On the latest CT, all the pulmonary lesions were completely absorbed, the duration of lesions was 31.6 ± 11.4 days (range: 5-50 days).

**Conclusion:** The pulmonary lesions in some mild COVID-19 patients (generally with normal laboratory indicators at admission, GGOs as the main manifestation on initial CT, and representation of continuous absorption after treatment) could be completely absorbed with a mean duration of 31.6 days.

## Introduction

Since December 2019, the occurrence of unexplained pneumonia was first reported in Wuhan City, Hubei Province, China 1. Virus detected and isolated from the patient's respiratory epithelial cells was named Severe Acute Respiratory Syndrome Coronavirus-2 (SARS-CoV-2). Subsequently, pneumonia caused by the infection of this virus was called Coronavirus 2019 (COVID-19). This virus is highly contagious, approximately with a basic regeneration number of 2.2 2. Currently, the COVID-19 has spread worldwide and was declared a global health emergency by WHO. As of April 20, 2020, more than 2 million cases have been confirmed in more than 200 countries and regions 3.

The SARS-CoV-2 mainly involves the respiratory system. Chest CT has a high sensitivity for diagnosing COVID-19 pneumonia 4, and plays an important role in the follow-up of COVID-19 patients. Due to positive findings on CT scans could be found before appearance of symptoms 5, thus chest CT also can be used for screening patients with negative reverse transcriptase polymerase chain reaction (RT-PCR) results in the early stage 6,7. Several studies on COVID-19 imaging have described the CT features of this disease and the evolution of the lesions 8-12. However, at present, there were no systematical reports on the outcome of this disease except a study reporting only one patient with complete absorption of the pulmonary lesion on the 31st day after the onset of symptoms 13.

In view of this, whether complete absorption of COVID-19 pneumonia is common, how long is the duration of lesions, and the clinical and imaging characteristics of those patients with completely absorbed lesions are unknown. Our hospital as one designated medical center for treating COVID-19 patients, a total of 224 patients were cured and discharged from hospital without death. After discharge, follow-up CT scans were still performed in our hospital. Currently, it was found that some mild patients' pulmonary lesions have been completely absorbed on repeat CT. This study aims to investigate their clinical and imaging characteristics for better understanding the outcome of this disease.

## Methods

This retrospective study used non-identifiable patient data for analysis and was approved by the institutional review board. Therefore, the requirement for informed consent was waived. The collection and analysis of anonymous data pose no potential risk to patients.

### Patients

A total of 224 patients with COVID-19 confirmed by RT-PCR test were admitted to our hospital from January 24, 2020 to March 27, 2020. Inclusion criteria: (1) there were pulmonary lesions on initial chest CT; (2) the patients' clinical data and laboratory examinations at admission were complete. Exclusion criteria: (1) the pulmonary lesions did not change or were absorbed incompletely on the latest repeat CT; (2) the interval between initial chest CT scan and hospitalization was more than 1 day.

### Treatment and discharge criteria

The treatment and discharge standards for COVID-19 patients were in compliance with the “Diagnosis and Treatment Program of Coronavirus Disease 2019 (Trial Seventh Edition)” 14. Discharge criteria were: (1) the body temperature returned to normal for more than 3 days, (2) respiratory symptoms improved significantly, (3) chest imaging examinations showed significant improvement of acute exudative lesions, (4) two consecutive nucleic acid tests on respiratory tract specimens were negative (the interval of two test must be at least 24 hours).

### CT protocol and follow-up CT scans

All patients underwent a non-contrast CT upon admission to the hospital. The initial and repeat chest CT scans were performed using Aquilion ONE (Toshiba Medical Systems, Tokyo, Japan) and Neusoft on-board 32-row CT scanner with the following parameters: tube voltage, 120 kV; automatic tube current modulation; beam pitch, 1.2/1.438; detector collimation, 0.625/0.6 mm; rotation time, 0.5 s; matrix size, 512 × 512; and section thickness and interval, 5.0 and 5.0 mm, respectively. All patients were in a supine position and scanned from the thoracic inlet to the lung base at the end of inspiration. CT images were reconstructed using a medium sharp reconstruction algorithm with a section thickness of 1 mm.

For monitoring the changes of pulmonary lesions in order to better understanding the improvement of disease, individualized follow-up CT scans were performed (interval: 3-5 days) based on the initial chest CT results, patients' clinical conditions, and the result of nucleic acid tests of SARS-CoV-2. After discharging from hospital, follow-up CT scans were performed at an interval of one or two weeks in the first month.

### Imaging analysis

Two senior radiologists (Lv and Chu), specialized in chest imaging read all the patients' first and follow-up CT images independently. The first step is to determine whether the pulmonary lesions in the initial chest CT scan are related to COVID-19. Subsequently, the follow-up CT data were reviewed and compared with the initial result for determining the changes and residues of lesions. The evaluation indicators are as follows: a. distribution of initial lesions (unilateral lung or bilateral lungs, involved lobes and segments); b. involved scope (the number of involved lobes and segments); c. the shape of lesions (round or patchy); d. density (ground glass opacity or consolidation); e. internal features (fibrous strips or consolidation); f. the changes of lesions one repeat CT (progression or directly absorption), g. outcome of lesions on latest CT scan (no change, incompletely absorption, complete absorption). In addition, pleural effusion and mediastinal and hilar lymphadenopathy were also evaluated. The result was based on the opinions of the two radiologist and reached an agreement.

### Clinical features

Clinical and laboratory data of the patients were collected by one radiologist (Liu). Clinical data, including age, gender, clinical type, initial symptoms, length of hospitalization, numbers of scans, and the interval between the initial and latest CT scans were recorded. Laboratory findings such as white blood cell count, neutrophil count and percentage, lymphocyte count and percentage, C-reactive protein, erythrocyte sedimentation rate, and lactate dehydrogenase, were also recorded. These laboratory tests were performed at admission and discharge.

### Statistical analysis

Statistical analysis was performed using SPSS (version 24; IBM, New York, USA). Continuous variables were expressed as mean ± standard deviation (minimum-maximum), and categorical variables were expressed as numbers and percentages. Independent sample t test (normal distribution or variance) or Mann-Whitney U test (non-normal distribution or variance) were used for statistical analysis. A p value < 0.05 was considered statistically significant.

## Results

### Clinical characteristics

Among the 224 patients with COVID-19, 37 (16.5%) cases were finally included in this study. The clinical characteristics of patients are shown in **Table 1**. The most common clinical manifestations were cough (64.9%) and fever (62.2%). The diseases in most patients were mild (97.3%), while that in the other one changed from severe into mild during treatment. During the absorption process, the clinical symptoms improved continuously in 29 (82.9%) cases and last for a relatively short period then improved in 6 (17.1%) cases among the 35 symptomatic patients.

### Laboratory indicators

The laboratory characteristics of the patients at admission and discharge are listed in **Table 2**. At admission, laboratory results in most patients were in normal limits, and most of those abnormal indicators recovered at discharge. The white blood cell count and neutrophil count at discharge were higher than those at admission (5.99 ± 1.84 vs. 4.83 ± 1.50, p = 0.004; 3.67 ± 1.43 vs. 2.81 ± 1.15, p = 0.006), while lactate dehydrogenase was significantly decreased at discharge compared with that at admission (164.14 ± 29.46 vs. 220.76 ± 90.64, p = 0.001).

In 37 patients, the average number of nucleic acid tests at the time of first turning negative and the total number of nucleic acid tests during hospitalization were 4.3 ± 4.1 (1 - 15) times and 5.7 ± 4.2 (range: 2-16) times, respectively. The mean time between the first positive nucleic acid test and the first negative nucleic acid test was 17.1 ± 8.8 (range: 5-36) days.

### CT features

At admission, all the 37 patients showed various pulmonary lesions on chest CT, their distribution and extent are shown in **Table 3**. The mean numbers of involved lobes and segments were 2.8 ± 1.5 (1-5) and 6.6 ± 4.3 (1-16), respectively. On CT images, pulmonary lesions manifested as multiple patchy and/or spherical GGOs in 30 (81.1%) cases (11 [36.7%] cases with little consolidation and 19 [63.3%] cases with fibrous strips), multiple patchy consolidations with little GGO in 4 (10.8%) cases; localized patchy GGOs with fibrous strips 3 (8.1%) cases. Besides these lesions, calcification, small solid nodules, fibrous strips, and bullae were found in 5 (13.5%) cases. No pleural effusion or mediastinal and hilar lymphadenopathy were detected in all patients.

The mean duration of hospitalization for the patients was 18.7 ± 8.2 days (range: 6-37 days). On repeat CT scans during treatment, lesions in 33 (89.2%) patients were continuously absorbed and in 4 (10.8%) cases showed a mild progression before absorption. At discharge, among the 37 patients, lesions in 11 (29.7%) cases were almost absorbed, in 25 (67.6%) showed residues of pure GGOs or GGOs with consolidation or fibrous strips, and in 1 (2.7%) case showed only few fibrous strips. The mean involved lobes and segments were 1.6 ± 1.6 (1-5) and 3.8 ± 4.1 (1-14) on the latest CT before discharge.

**Figure 1:**
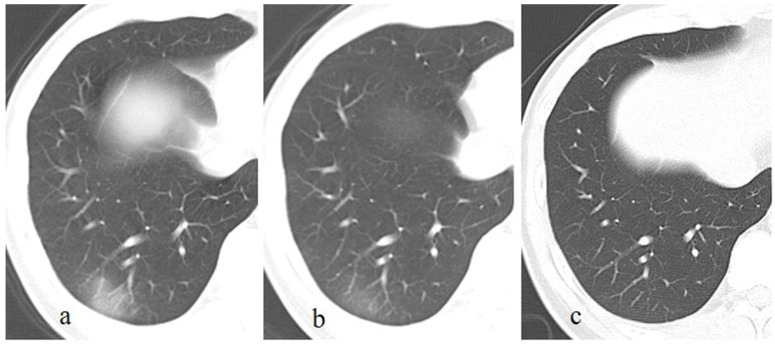
A patient with a history of travelling to Wuhan recently, presents with fever and cough for 13 days. The initial chest CT at the first day after admission shows a patchy GGO in the right lower lobe (a). Subsequently (5 days later), this lesion is directly absorbed with decrease of extent and density (b). On the latest CT scan (18 days later), the lesion is completely absorbed (c).

**Figure 2:**
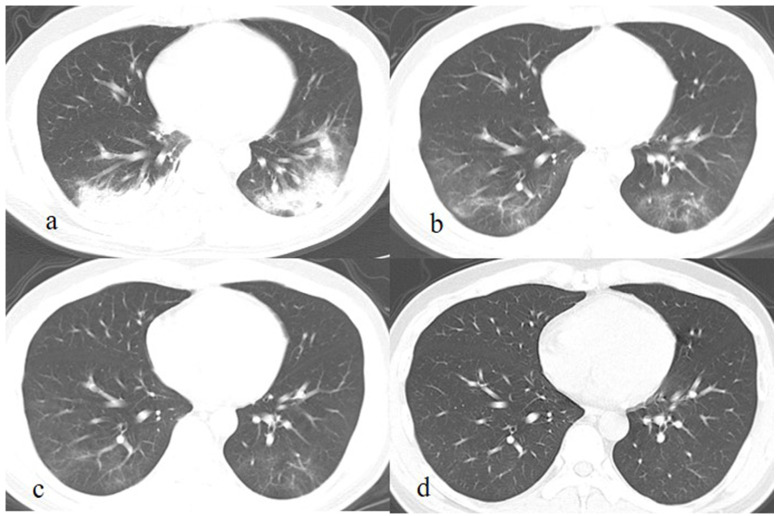
A patient exposed to COVID-19 presents with fever, cough, and fatigue for 7 days. The initial chest CT at the first day after admission shows patchy consolidations and GGO in the left and right lower lobes (a). Subsequently (7 and 13 days later), the lesions are continuously absorbed with decrease of extent and density, and fibrous strips are detected in lesions (b, c). On the latest CT scan (36 days later), the lesion is completely absorbed GGO (c).

After discharge, all the pulmonary lesions were completely absorbed on the latest CT (**Fig. 1-2**), the duration of lesions from occurrence to disappearance was 31.6 ± 11.4 days (range: 5-50 days). Among different lesions, duration of GGO was the longest (mean: 29.8 ± 12.1 days, range: 5.0-50.0), which was significantly longer than that of fibrous strips (mean: 23.8 ± 9.5 days, range: 12.0-44.0) (p = 0.046) and consolidation (mean: 9.4 ± 3.6 days, range: 4.0-15.0) (p = 0.000).

## Discussion

SARS-CoV-2 is the seventh member of the coronaviridae family known to infect humans, which is significantly different from SARS-CoV and MERS-CoV 14. In view of the current epidemic situation, SARS-CoV-2 is highly contagious, which posed a major threat to public health. However, most COVID-19 patients in our hospital showed a good prognosis, and pulmonary lesions in some cases were found to be completely absorbed in a short term, which was consist with the findings in another studies 15, 16. Those patients with completely absorbed lesions were usually younger and had normal laboratory indicators at admission, their disease were mainly mild, the mean duration of lesions was 31.6 days but the range was large, the lesions on initial CT were mainly manifested as GGOs and which were frequently absorbed continuously after treatment. To a certain extent, these results are helpful for better understanding this disease.

For SARS-CoV-2, the population is generally susceptible, and usually the elderly or those with underlying diseases are more severe after infection 17. In the present study, the average age of patients was 39 years old, which was lower than that of general patients in previous studies 12. It indicated that pulmonary lesions in younger patients were absorbed more quickly 15, which may be due to they have relative stronger immunity and less basic diseases. Laboratory examinations also revealed routine hematological and inflammatory indicators in most patients were normal or mildly abnormal at admission and those abnormalities usually return to normal before discharge. Additionally, the disease in most patients was mild, indicating lung injury was not serious 9, which may be also closely related to their better prognosis. Therefore, the pulmonary lesions in most patients may be completely absorbed finally because of the higher percentage of mild disease in infected cases 18.

Besides the clinical and laboratory characteristics, CT manifestations of pulmonary lesions and their changes also showed some characteristics during treatment. In this study, most lesions manifested as GGOs without significant consolidation at admission, and their extent was less than that in severe patients reported in previous study 19. After accepting treatment, lesions in most patients showed continuous absorption, which was different from those showing progression before absorption 8,11. These characteristics indicated the pulmonary damage was not as serious as that caused by significant consolidation 20, and the disease was well controlled. Correspondingly, no one symptomatic patient's clinical symptoms were worsening during treatment, which was consist with the radiological improvement. Though the clinical manifestations and radiological abnormalities were not significant in those patients, pulmonary function should be evaluated and long-term respiratory follow-up was needed because abnormal pulmonary function was prevalent in COVID-19 survivors 21, 22.

At present, the relationship between occurrence of fibrous strips and prognosis of COVID-19 patients is still controversial. Zhou S et al. 11 believed that the lesions could lead to pulmonary interstitial fibrosis after the appearance of fibrosis strips, indicating a poor prognosis. While Pan Y et al. 5 reported that the appearance of fibrous strips suggested improvement of disease and good prognosis. In the present study, the newly developed fibrous strips were commonly detected in pulmonary lesions during treatment, and most of which were completely absorbed eventually before GGO. Therefore, the appearance of fibrous strips may be a common manifestation in later stage of disease and it does not necessarily indicate a poor prognosis.

In contrast to the present results, a recent study reported the long-term lung radiographic changes in convalescent severe COVID-19 patients 23. On six-month follow-up, the existence of residual CT abnormalities in a large proportion (62%) of patients with severe COVID‐19 infection. In particular, the residual fibrotic-like changes were closely associated with an older age, acute respiratory distress syndrome, longer in-hospital stays, tachycardia, non-invasive mechanical ventilation and higher initial chest CT score. Thus, radiological resolution of pulmonary lesions in COVID-19 patients is related to many factors, which is relatively faster in mild cases while the final conditions of residual CT abnormalities in severe cases needs long-term follow-up.

The time from the onset of lesions to complete absorption varied among individuals with a large range. Absorption of GGOs was significantly slower than that of fibrous strips or consolidation in the present and previous study 16. The pathological basis for GGO caused by SARS-Cov-2 included alveolar injury, edema, and exudation with alveolar epithelial cell proliferation and inflammatory infiltration 24. Therefore, the duration of GGO or pulmonary lesions was closely related to the degree of pulmonary injury. Longer duration of lesions represented more serious injury.

There are the four limitations in this study. First, the number of subjects in final analysis was relatively small. When we conducted this study, pulmonary lesions were completely absorbed only in 37 patients because the patients were admitted to hospital in different stages. Second, all the patients had pulmonary lesions on initial CT scan and there were intervals between two repeat CT scans, thus the exact duration of lesions could not be acquired. Third, we did not compare the patients with complete and incomplete absorption of lesions at the same period. The cases in control group cannot be well matched because the duration of lesions significantly varied among patients. Fourth, disease in most patients in the present study was mild. The patients with severe disease were not included because they still had residues on the latest CT scan though the lesions were continuously absorbed. Thus, this is a preliminary study; further studies with large samples are needed for better understanding the clinical and radiological changes of COVID-19 patients with different severity.

## Conclusions

In conclusion, pulmonary lesions in some patients with mild COVID-19 pneumonia could be completely absorbed in a short term, with a mean duration of 31.6 days and a large range. These patients are usually younger and have normal laboratory indicators at admission. Their lesions on initial CT are mainly manifested as GGOs, and which frequently be absorbed continuously after treatment.

## Figures and Tables

**Table 1:** The clinical characteristics of patients

Parameters	Value
**Gender**	
Male	20 (54.1)
Female	17 (45.9)
Age (years)	39.0 ± 12.4 (14 - 63)
**Time of onset of symptoms**	
Average (days)	4.6 ± 3.0 (1-12)
≥7 days	29 (78.4)
< 7 days	6 (16.2)
**Exposure History**	
Recently travel to main epidemic area	10 (27.0)
Exposure to infected patients	19 (51.4)
Contacting with people from epidemic area	4 (10.8)
Unknow	4 (10.8)
**Clinical type**	
Mild	36 (97.3)
Severe	1 (2.7)
**Maximum body temperature (°C)**	
37.3-38.0	17 (45.9)
38.1-39.0	6 (16.2)
>39.0	0 (0)
**Clinical manifestations**	
Fever	23 (62.2)
Cough	24 (64.9)
Sputum	11 (29.7)
Headache	7 (18.9)
Sore Throat	7 (18.9)
Fatigue	6 (16.2)
Diarrhea	6 (16.2)
Muscle Soreness	4 (10.8)
Chills	3 (8.1)
Asymptomatic	2 (5.4)

Data are expressed as mean ± standard deviation (range) or n (%).

**Table 2:** The laboratory characteristics of patients

Laboratory examinations	At admission	At discharge
**White blood cell count (G/L)**		
Normal/decreased	33 (89.2) / 4 (10.8)	34 (91.9) / 3 (8.1)
Neutrophil count (G/L)		
Normal/increased/decreased	32 (86.5)/ 0 (0)/ 5 (13.5)	33 (89.2)/ 2 (5.4) /2 (5.4)
**Neutrophil percentage (%)**		
Normal/increased/decreased	32 (86.5) / 2 (5.4) / 3 (8.1)	36 (97.3) / 0 (0) / 1 (2.7)
Lymphocyte count (G/L		
Normal/increased/decreased	32 (86.5) / 0 (0) / 5 (13.5)	33 (89.2) / 1 (2.7) / 3 (8.1)
**Lymphocyte percentage (%)**		
Normal/increased/decreased	32 (86.5) / 2 (5.4) / 3 (8.1)	35 (94.6) / 0 (0) / 2 (5.4)
**C-reactive protein (mg/L)**		
Normal/increased	20^a^ (66.7) / 10^a^ (33.3)	18^b^ (85.7) / 3^b^ (14.3)
**Lactate dehydrogenase (U/L)**		
Normal/increased	27 (73.0) / 10 (27.0)	35^c^ (100.0) / 0^c^ (0.0)
CD3 (/μL)		
Normal/decreased	28 (75.7) / 9 (24.3)	8^d^ (80.0) / 2^d^ (20.0)
CD4 (/μL)		
Normal/decreased	25 (67.6) / 12 (32.4)	8^d^ (80.0) / 2^d^ (20.0)
CD8 (/μL)		
Normal/decreased	29 (78.4) / 8 (21.6)	9^d^ (90.0) / 1^d^ (10.0)

Notes: ^a^, n = 30; ^b^, n = 21; ^c^, n = 35; ^d^, n = 10 Data are expressed as n (%).

**Table 3:** Distribution and extent of pulmonary lesions at admission

Distribution and extent	Value
Bilateral lung involvement	27 (73.0)
Unilateral lung involvement	10 (27.0)
**Number of involved lobes**	
1	10 (27.0)
2	6 (16.2)
3	7 (18.9)
4	8 (21.6)
5	6 (16.2)
**Frequency of involved lobes**	
Right upper lobe	14 (37.8)
Right middle lobe	14 (37.8)
Right lower lobe	32 (86.5)
Left upper lobe	17 (45.9)
Left lower lobe	28 (75.7)
**Number of involved segments**	
1	2 (5.4)
2-4	13 (35.1)
5-7	9 (24.3)
8-10	5 (13.5)
>11	8 (21.6)

Notes: data are expressed as n (percentage).

## Abbreviations

COVID-19: coronavirus disease 2019
GGOs: ground-glass opacities
RT-PCR: reverse transcriptase polymerase chain reaction
